# Supplementary material for: SmartScope2: Simultaneous Imaging and Reconstruction of Neuronal Morphology
Source: Sci Rep. 2017 Aug 24;7:9325. doi: 10.1038/s41598-017-10067-w (PMC5571186; doi:10.1038/s41598-017-10067-w)
Supplement: Supplementary file 1 — Supplemental Information [file 41598_2017_10067_MOESM1_ESM.doc]

# SmartScope2: Simultaneous Imaging and Reconstruction of Neuronal Morphology

Brian Long, Zhi Zhou, Ali Cetin, Jonathan Ting, Ryder Gwinn, Bosiljka Tasic, Tanya Daigle, Ed Lein, Hongkui Zeng, Peter Saggau, Michael Hawrylycz, and Hanchuan Peng

**Supplemental Figures and Text**


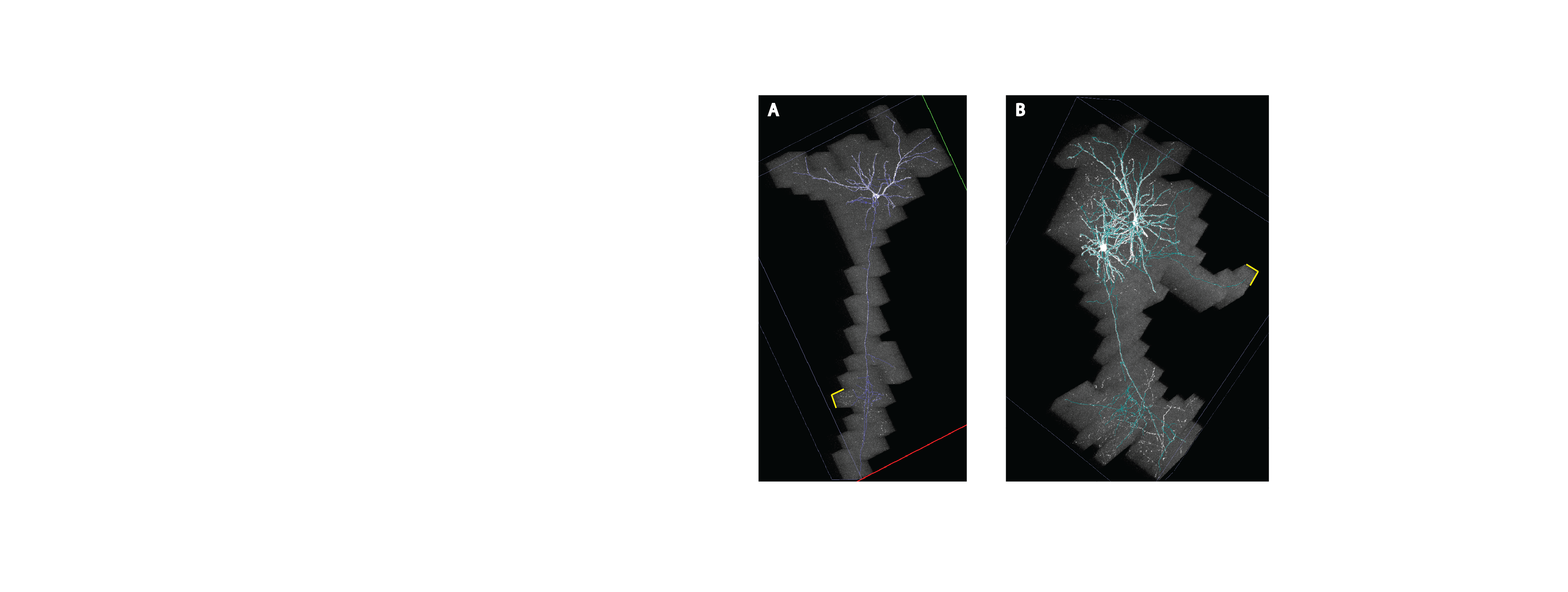


Figure S1. Additional S2 scans 3 (panel A) and 6 (panel B) referred to in Table 1. Yellow scale bars are 36 µm.


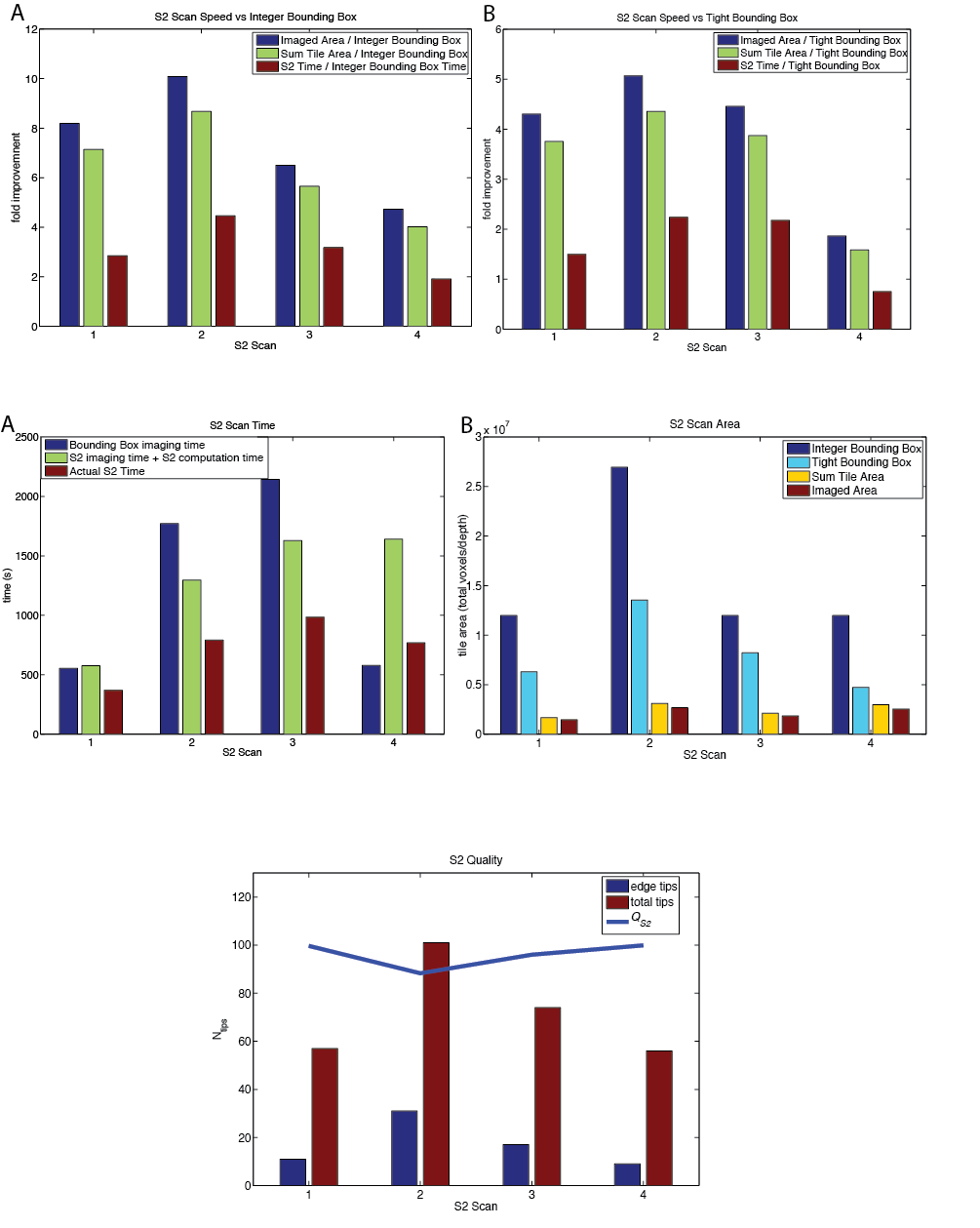


Figure S2. S2 scan improves speed and image data for isolated neuron reconstructions. A. Improvement of S2 scanning area (blue), sum of S2 tiles (green) and actual imaging time (maroon), compared to the rectangular bounding box of maximum-area tiles. B. Scan speed improvements and area improvements of S2 scan in comparison to the tight rectangular bounding box- the minimal rectangular scan that would contain all of the imaged tiles.


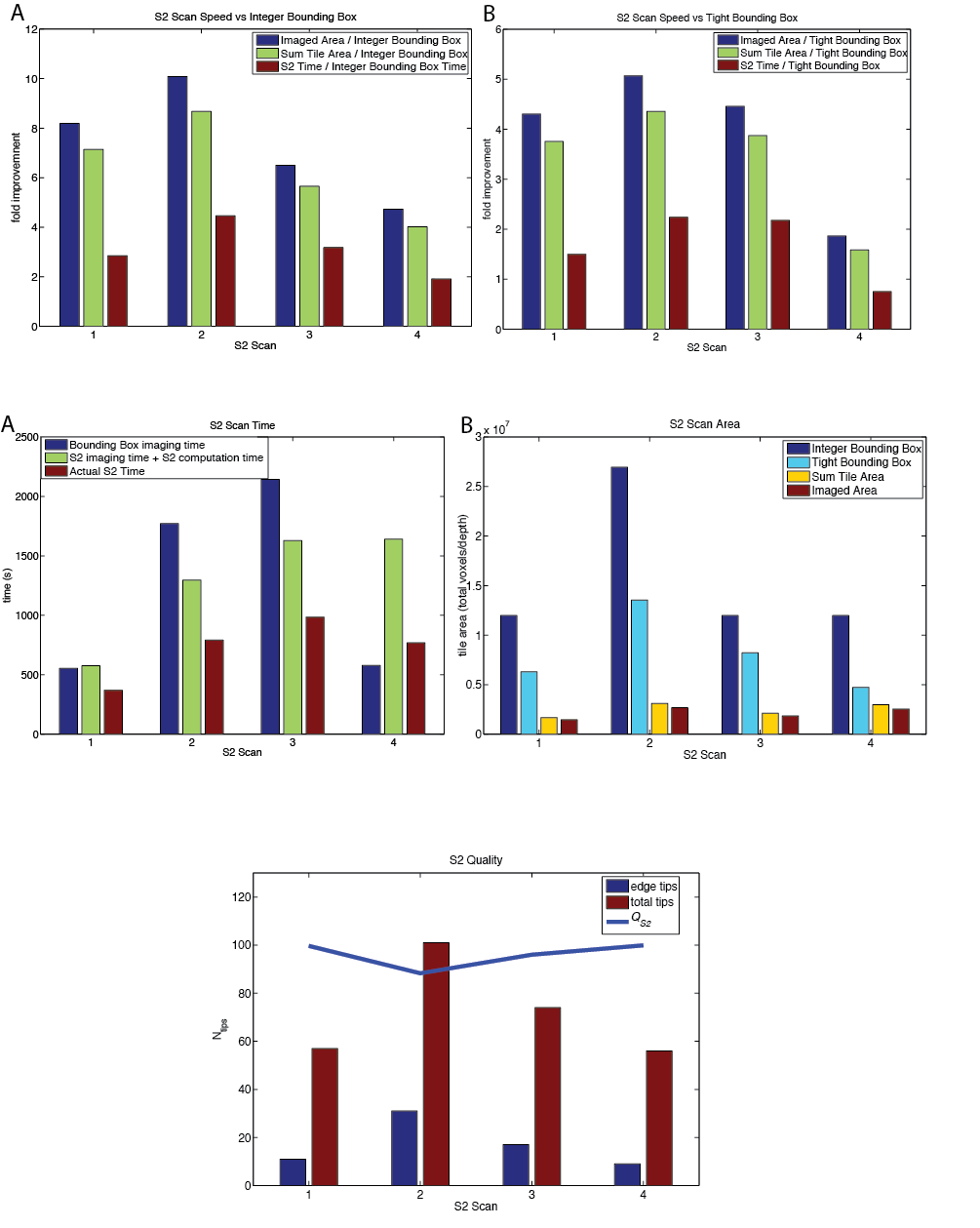


Figure S3. A. Scan times for S2 scans (red), S2 imaging + S2 analysis times (green) are the combined imaging and computation times, which would represent the total time if S2 did not use asynchronous data acquisition and multithreaded reconstruction. B. Total area in voxels for isolated neurons 1-4, showing integer bounding box and tight bounding box areas, as well as the sum of all imaged tiles and the total imaged area for S2 scans.


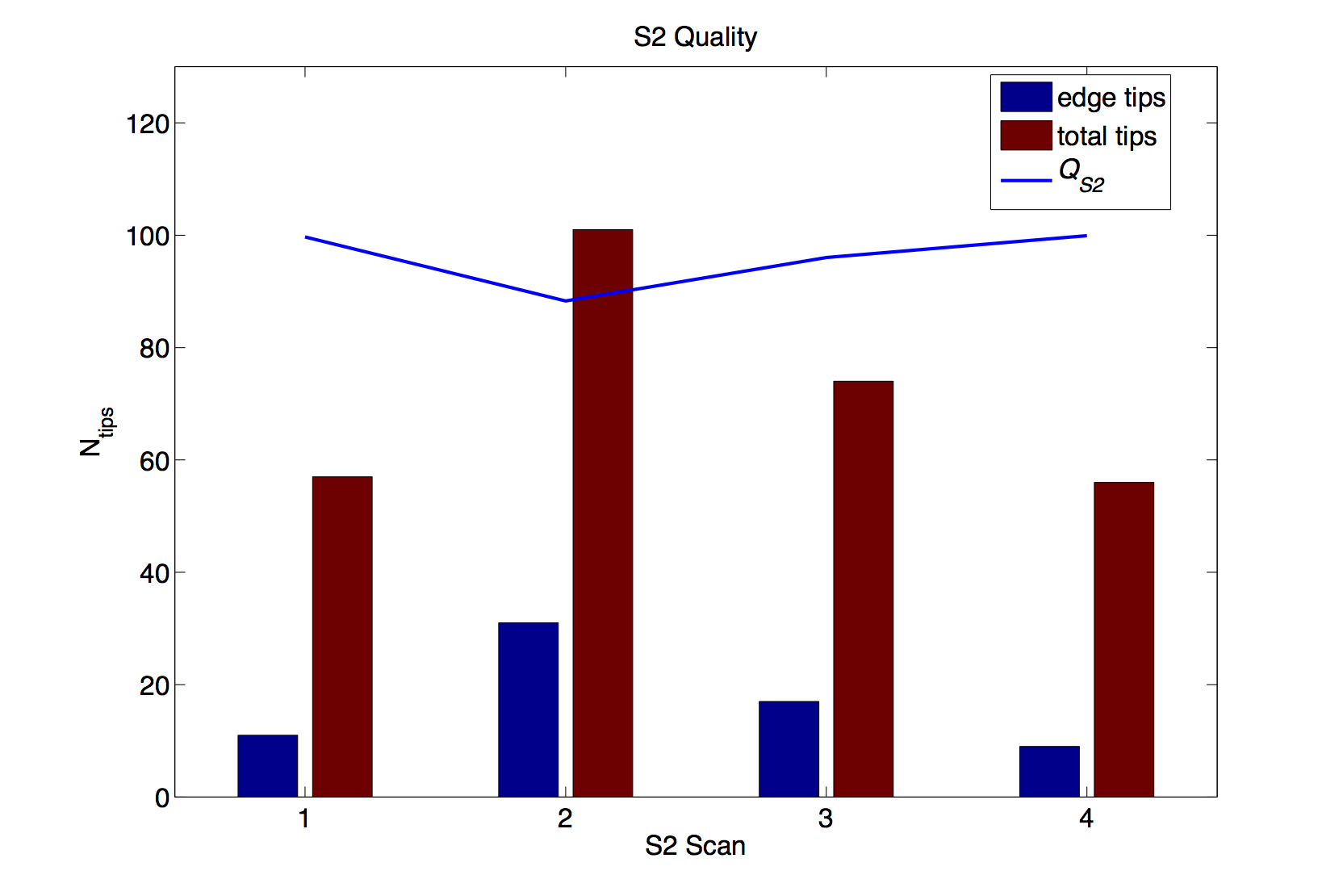


Figure S4 S2 quality index (blue line) and bar plot showing the number of reconstruction tips within 5% of the edge of a tile (blue bars) and the total number of tips for each reconstruction (maroon bars).

| Scan Number | 1 | | 2 | | 3 | | 4 | | 5 | | 6 | | 7 | |
| --- | --- | --- | --- | --- | --- | --- | --- | --- | --- | --- | --- | --- | --- | --- |
| Cell Type | Mouse SS 2/3 | | Mouse SS Layer 5 | | Mouse SS Layer 2/3 | | Human MTG Layer 2 | | Mouse SS Multi-neuron Scans | | | | | |
| Imaged Area (voxels) | 1.46E+06 | | 2.67E+06 | | 1.84E+06 | | 2.53E+06 | | 3.71E+06 | | 2.99E+06 | | 1.03E+07 | |
| Sum of Tile Areas (voxels) | 1.68E+06 | | 3.11E+06 | | 2.12E+06 | | 2.98E+06 | | 4.24E+06 | | 3.52E+06 | | 1.24E+07 | |
| X_TightBB (µm) (voxels) | 424 | 1850 | 875 | 3823 | 553 | 2414 | 423 | 1849 | 972 | 4246 | 746 | 3259 | 1199 | 5234 |
| Y_TightBB (µm) (voxels) | 779 | 3401 | 811 | 3541 | 779 | 3400 | 585 | 2555 | 1360 | 5938 | 617 | 2696 | 1586 | 6926 |
| X_Int. BB (µm) (voxels) | 792 | 3460 | 1189 | 5190 | 792 | 3460 | 792 | 3460 | 1189 | 5190 | 792 | 3460 | 1585 | 6920 |
| Y_Int. BB (µm) (voxels) | 792 | 3460 | 1189 | 5190 | 792 | 3460 | 792 | 3460 | 1585 | 6920 | 792 | 3460 | 1981 | 8650 |
| Area Efficiency | 7.1 | | 8.7 | | 5.6 | | 4.0 | | 8.5 | | 3.4 | | 4.8 | |
| S2 Speedup (int. BB) | 2.8 | | 4.5 | | 3.2 | | 1.9 | | 3.7 | | 2.0 | | 1.3 | |
| S2 Speedup (tight BB) | 1.5 | | 2.2 | | 2.2 | | 0.8 | | 2.6 | | 1.4 | | 0.8 | |
| n tiles | 68 | | 126 | | 86 | | 120 | | 176 | | 142 | | 504 | |
| n_z planes | 169 | | 251 | | 501 | | 235 | | 191 | | 457 | | 213 | |
| z step (µm) | 1 | | 1 | | 0.5 | | 1 | | 1 | | 0.5 | | 1 | |
| total s2 time (s) | 370 | | 791 | | 984 | | 768 | | 968 | | 1454 | | 5009 | |
| total imaging time (s) | 330 | | 768 | | 867 | | 726 | | 901 | | 1383 | | 4888 | |
| total analysis time (s) | 246 | | 529 | | 761 | | 914 | | 544 | | 1919 | | 7807 | |
| number of tips | 57 | | 101 | | 74 | | 56 | | 123 | | 167 | | 1328 | |
| number of edge tips | 11 | | 31 | | 17 | | 9 | | 46 | | 72 | | 516 | |
| expected number of tips | 10 | | 19 | | 14 | | 10 | | 23 | | 31 | | 252.3 | |
| Q_S2 | 0.98 | | 0.88 | | 0.96 | | 1.02 | | 0.81 | | 0.75 | | 0.80 | |

Table S1. S2 scan characteristics for four isolated neurons (1-4) from mouse and human (as indicated) and three multi-neuron scans (5-7) from somatosensory cortex (SS). Area efficiency listed here compares the area of the max-field bounding box (“Int. BB”) to the summed area of all S2 tiles. The tile size is 36 µm on a side: 157x157 pixels with 0.23 µm pixel size.

**S2 Scan: Pilot Dataset information**

These data were collected from neurons labeled via sparse recombination of Cre in an EGFP-tdTomato reporter line (Ai139). The S2 scan was accomplished as described in the main text but using nonresonant scanning and galvo-based ROI steering instead of xy stage movement. As seen in Figure S3, S2 achieved sparse imaging, but square bounding box imaging was generally faster than the S2 scan in this pilot study due to the increasing time per pixel for smaller scan size. This pilot study was able to achieve small tile sizes by using galvo-based ROI steering and nonresonant scanning, but the increased imaging time per pixel for small tile sizes compared to large scans reduced the speed benefit of S2. However, S2 scanning still afforded a sparsity factor of 0.28 ± 0.096 (N=20) compared to a square bounding box. For the data reported in Table S1, where we used resonant scanning as described in Methods in the main text, microscope scan setup time, communication between S2 and the microscope control software and image data transfers resulted in ~1.5s overhead per tile, corresponding to an increase in effective imaging time per pixel between S2 tiles and maximum-area tiles of roughly 25%.


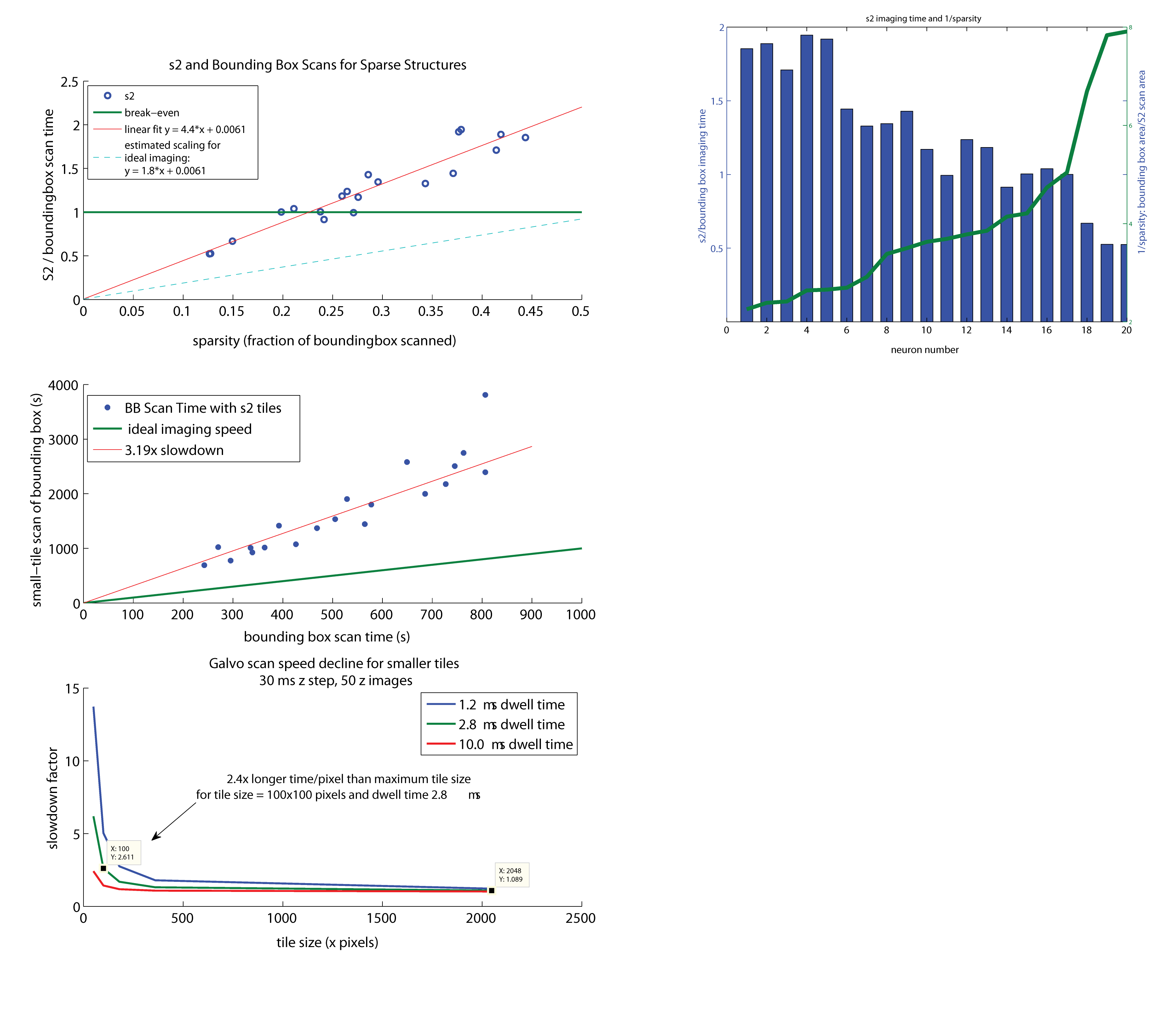


Figure S5. S2 time for 20 neurons, normalized to experimental square bounding box scan time for the same neuron. The square bounding box here is defined as the minimal square area that contained all of the S2 tiles. This square bounding box was scanned to determine the bounding box imaging time, to which the s2 scan time is normalized (left axis). Neurons are ordered by increasing values of 1/sparsity, which is plotted in green (right axis).

**Manual Reconstruction and Distance Score:**

To evaluate the accuracy of S2 reconstructions, three annotators manually traced neurons in the S2 scans from three isolated mouse neurons (M1, M2, and M3 in Table S2) using Virtual Finger^1^ through the Vaa3D-Terafly^2^ interface. To quantify reconstruction similarity, we use the “best average spatial distance score”, defined as the average of the shortest distance between compartments in two reconstructions^3^. Table S2 shows the pairwise comparison results between annotators and the S2 reconstructions.
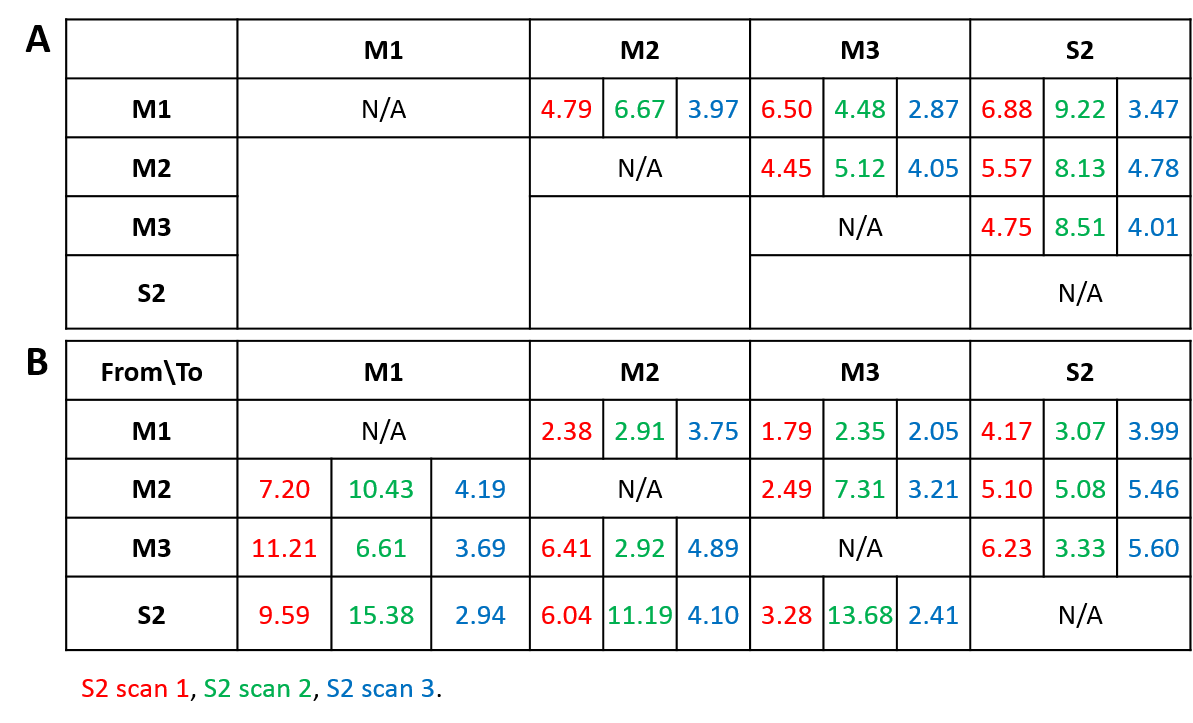


Table S2. A. The best average spatial distance score between two reconstructions (average of bi-directional score). B. The best average spatial distance from one reconstruction to another (directional score), Distance scores on three different isolated mouse neurons (Table S1) have been marked as red (scan 1), green (scan 2), and blue (scan 3).


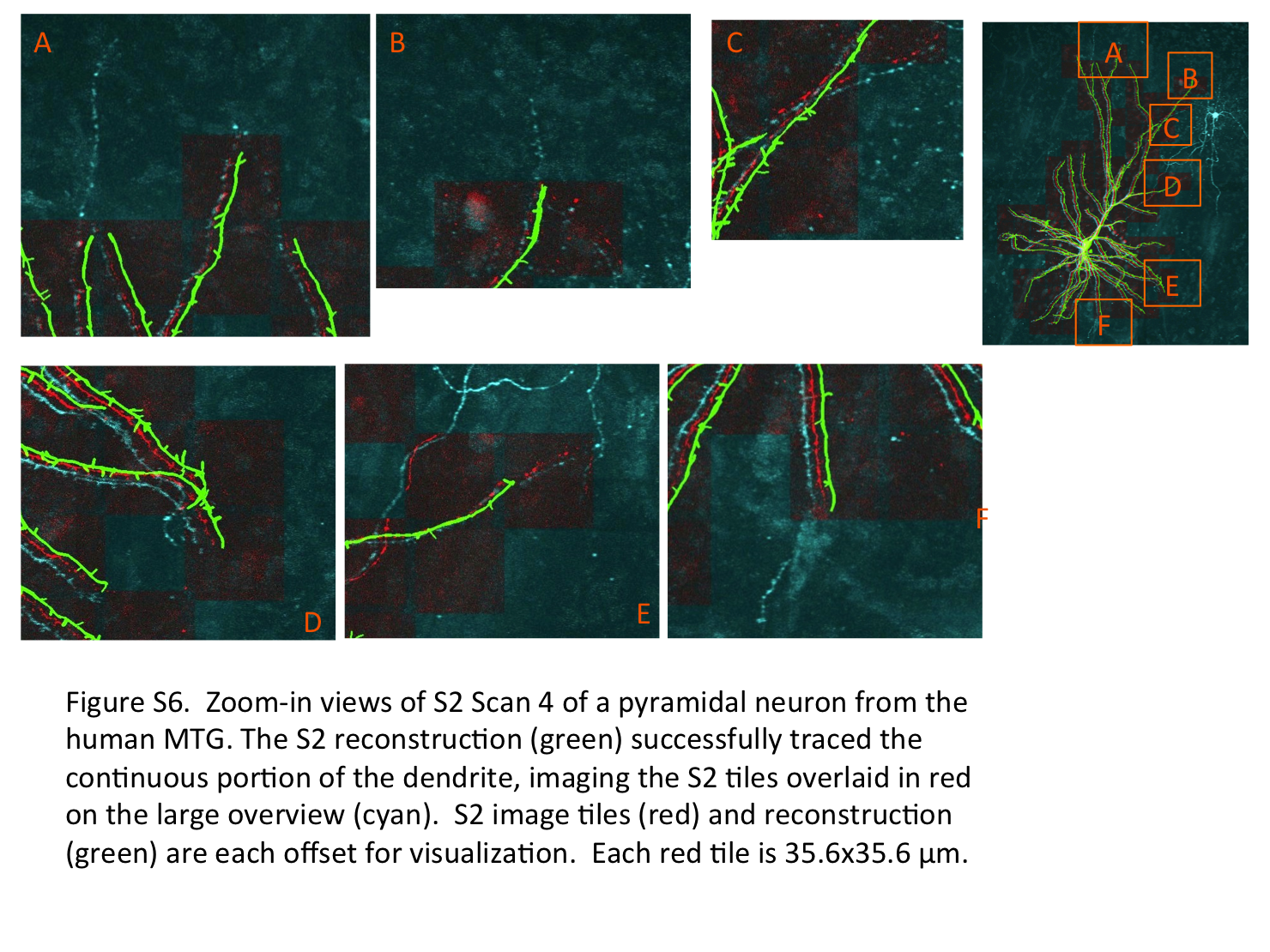


Figure S6. Zoom-in views of S2 Scan 4 of a pyramidal neuron from the human MTG, showing the 6 locations where the S2 reconstruction terminated on weak or discontinuous signal, out of 56 tips. The S2 reconstruction (green), imaging the S2 tiles overlaid in red on the large overview (cyan). S2 image tiles (red) and reconstruction (green) are each offset for visualization. Each red tile is 35.6 x 35.6 x 235 µm.


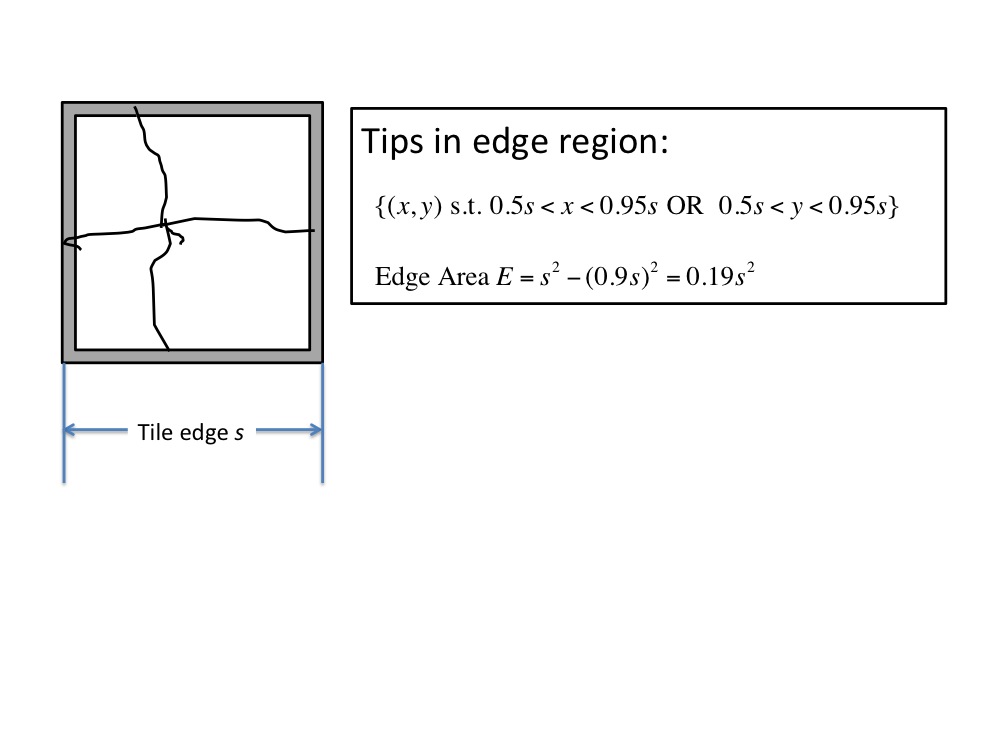


Figure S7. Illustration of ‘edge tips’ used in S2 Quality index. For edge regions defined as within 5% of the tile border, 19% of randomly distributed tip locations would be expected to be counted as edge tips.

**Supplementary References:**

1. Peng, H. et al. Virtual finger boosts three-dimensional imaging and microsurgery as well as terabyte volume image visualization and analysis. Nat. Commun. 5, 4342 (2014).

2. Bria, A., Iannello, G., Onofri, L. & Peng, H. TeraFly: real-time three-dimensional visualization and annotation of terabytes of multidimensional volumetric images. Nat. Methods 13, 192–194 (2016).

3. Peng, H., Ruan, Z., Atasoy, D. & Sternson, S. Automatic reconstruction of 3D neuron structures using a graph-augmented deformable model. Bioinforma. Oxf. Engl. 26, i38-46 (2010).
